# The Genus *Neoceratium* (Planktonic Dinoflagellates) as a Potential Indicator of Ocean Warming

**DOI:** 10.3390/microorganisms1010058

**Published:** 2013-10-25

**Authors:** Alina Tunin-Ley, Rodolphe Lemée

**Affiliations:** 1 Université Pierre et Marie Curie-Paris 6, Villefranche Oceanographic Laboratory, BP 28, 06234 Villefranche-sur-Mer cedex, France; 2 CNRS (Centre National de la Recherche Scientifique), Marine Microbial Ecology and Biogeochemistry Group, Villefranche Oceanographic Laboratory, BP 28, 06234 Villefranche-sur-Mer cedex, France; 3 ARVAM (Agence pour la Recherche et la VAlorisation Marines), C/o CYROI., 2 Rue Maxime Rivière, 97490 Sainte Clotilde, France; E-Mail: alina.tunin-ley@arvam.com

**Keywords:** *Neoceratium*, *Ceratium*, ocean warming, dinoflagellate, biological indicator

## Abstract

Among the planktonic dinoflagellates, the species-rich genus *Neoceratium* has particularly remarkable features that include its easily recognizable outline and large size. This ubiquitous genus shows consistent presence in all plankton samples and has been a model for numerous studies since the end of the 19th century. It has already been described as a good candidate to monitor water masses and describe ocean circulation. We argue that the sensitivity displayed by *Neoceratium* to water temperature also makes it relevant as an indicator of ocean warming. The advantages and interests of using *Neoceratium* species to monitor climate change on a large scale are reassessed in view of recent advances in understanding their biology and ecology.

## 1. A Species-Rich Genus of Historical Importance

Most scientists who have had the opportunity to observe marine phytoplankton under a microscope describe *Ceratium* as curious anchor-shaped or three-horned organisms. Of the dinoflagellates, the most famous genera are probably *Ceratium* Schrank and *Protoperidinium* Bergh, emend Balech, 1974, the history of which is very similar. As for *Protoperidinium*, the first descriptions of *Ceratium* taxa are very ancient and for both genera, the marine species have only recently been separated from freshwater ones and grouped under a different genus name, after studies provided morphological evidence favoring such a division [1]. Thus, the new genus name, *Neoceratium*, proposed by Gómez *et al.* [2], identifies the marine species that once belonged to the genus *Ceratium*.

Prior to 1987, most bibliographic records correspond to studies focusing directly on *Neoceratium*. An observed increase in the number of records during the last 25 years of the 20th century likely corresponded more to an increasing trend in the number of publications in all fields. Indeed, numerous studies that both do or do not focus on phytoplankton, mention, cite or list *Neoceratium* species (Table 1).

**Table 1:** Results of Web of Knowledge bibliographic search for “*Neoceratium*” or “*Ceratium*” entries within the title or main text.

Number of Bibliographic Records for *Neoceratium* or *Ceratium* Entries		
Period	Title	Main text
1950–1987	54	55
1988–2013	80	399
Total	134	454

Two remarkable features could explain the recurrent citations of *Neoceratium*: worldwide distribution and species richness. The ubiquitous presence of this genus within the equatorial seas up to the polar ecosystems, in oceanic as well as neritic waters [3,4], explain why *Neoceratium* species are commonly observed in phytoplankton samples. Indeed, whatever the sampling methodology, absence of *Neoceratium* species within the microplankton sample is extremely rare. In addition the genus is characterized by a remarkably specific and also infraspecific richness [3] and includes more than 120 morphological taxa describing different species, infraspecific forms and varieties [5] that exhibit a great variety of shapes. As fairly large cells (from around 50 μm long up to 1 mm) that are typically outlined and widely distributed, they were logically among the very first described taxa of the microplankton.

**Figure 1:**
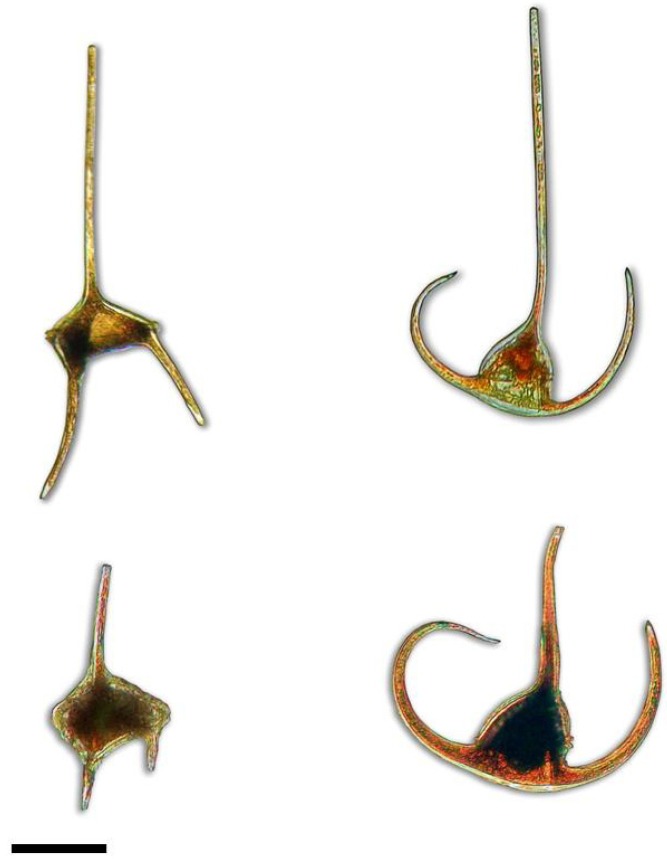
Illustration of infraspecific morphological variations in *Neoceratium* species. Slender variety *depressum* (upper left) and robust variety *candelabrum* (bottom left) in *N. candelabrum*. Slender variety *gracilentum* (upper right) and robust variety *arietinum* (bottom left) in *N. arietinum*. Bar scale 50 μm. Lugol-fixed cells.

The first illustrations of *Neoceratium* species by Müller [6] date from the end of the 18th century and the first monograph of the genus was provided by Jörgensen in 1911 [7]. Since then, numerous studies and descriptions of *Neoceratium* taxa have followed leading to profuse literature but also an inextricable jumble of taxonomic designations. Indeed, while some authors interpreted the high morphological variability as criteria for species delineation, others viewed it as the expression of infraspecific variability. This resulted in a multiplication of taxonomic names and synonyms. In the 1980s, Sournia considered that there were 120 reliable names, 85 uncertain names and as many invalid names of infraspecific taxa [8]. This observation led to the author proposing a non-conventional nomenclature in his own monograph of the genus, to identify and characterize the high morphological variability with the most plausible accuracy [3]. From his observations and the analysis of former studies, he suggested that water temperature was a constraining factor for *Neoceratium* species, and that the morphological variability observed within the genus may be explained by the environmental conditions resulting from the effect of temperature on the physical property of water, *i.e.*, viscosity of the medium. Indeed, for several *Neoceratium* species, certain cells exhibit a slender general aspect traducing a trend to extension (thin and long horns, and the occurrence of numerous expansions or inflated horns) whereas others show a robust outline (shorter and wider horns and central body, the occurrence of crests, and thicker theca) tending towards compactness. The slender forms may thus reflect an adaptation of a species to improve their floatability in warmer waters characterized by lower viscosity. On the other hand, the higher viscosity of colder waters would offer better floatability to cells thus allowing the development of compact and robust forms (Figure 1). According to Sournia, the analysis of the seasonal and biogeographical occurrence of several taxa based on the compilation of several observations corroborates this hypothesis and supports the infraspecific level of the morphological variability in *Neoceratium* genus [3]. To clarify the taxonomic identifications within this genus, he developed a totally new nomenclature which reconsidered several species as extreme infraspecific forms adapted to opposite temperature conditions, and characterized cells with intermediate shapes which seemed to constitute transition adaptations between the extreme varieties of a species (see Sournia for nomenclature criteria [3]). Although this nomenclature has been rarely used since, it nevertheless provides a very useful tool to describe without confusion the amazing morphological variability in this genus, and represents a first attempt to relate the taxonomic descriptions in the literature to each other and clarify the corresponding taxonomic designations.

## 2. A Frequent Model in Marine Research Studies

Its frequent occurrence in water samples has made *Neoceratium* one of the preferred models in studies focusing on phytoplankton, in particular experimental studies investigating different aspects of dinoflagellate ecophysiology, including their bioluminescence [9,10], flagella mobility [11,12], cell division and growth rate [13,14,15], diurnal cycle [16], trophic relationships and mixotrophy [17,18,19]. The large size of the cells and the characteristic morphological features of the horns in *Neoceratium* constitute a great advantage in terms of enabling easy discrimination and isolation from a sample. Abundance, frequency and easy recognition are all important factors to consider when choosing a biological model as together they ensure a constant supply of cells and permit the reproduction of experiments and measurements. Accordingly, *Neoceratium* represents an ideal candidate as a biological model.

*Neoceratium* is also an interesting model for studying water masses and current regimes. For instance, analysis of the valuable time-series provided by the Continuous Plankton Recorder in the North Atlantic Sea has revealed a strong relationship between the occurrence of dinoflagellates, including numerous *Neoceratium* species, and the description of the water circulation [20]. In the Pacific Ocean, the distribution patterns of dinoflagellates appear to reflect the development of El Niño events [21] as some species are indicators of different water masses affected by this phenomenon. One example is *Neoceratium breve* which is considered as an indicator of Equatorial Surface Waters [21,22].

In addition, several biogeographical investigations have confirmed that the majority of *Neoceratium* species are restricted to marine regions characterized by specific thermal conditions [4,23]. A large study performed in the North Atlantic Ocean based on numerous observations and bibliographic data proposed the categorization of the species into six groups presenting different biogeographical distributions and thermal affinities [4]: an arctic-temperate group subjugated to temperatures less than 15 °C, a cosmopolitan group made up of the ubiquitous and frequently bloom-forming species, an intermediate group with species absent from the coldest and warmest waters, a temperate-tropical group subjugated to a lower temperature limit of 5–12 °C, a warm-temperate-tropical group with a lower thermic boundary of 14–15 °C, and a tropical group within which species are rarely found in waters with temperatures below 20 °C. A similar study based on the same approach was also conducted in the western Pacific Ocean but the multivariate analysis did not allow the constitution of logical groups of species, although biogeographical zones for *Neoceratium* could be defined by the analysis of sampling stations [24]. In the Arctic Ocean, one *Neoceratium* species appears to be one of the rare dinoflagellates that can be considered as Arctic-boreal taxa [25].

## 3. Investigating the Potential of *Neoceratium* Species as Indicators of Ocean Warming

Together, the features describing *Neoceratium*, *i.e.*, ubiquity, frequency, taxonomic richness and sensitivity to temperature, suggest this genus may provide interesting ecological indicators to monitor global warming. However, some prerequisites are needed to justify the relevancy of an ecological indicator. The selection of ecological indicators should ensure their meeting several criteria. They should be easy to measure, have high sensitivity and known response to stress, have predictable responses to stress, be anticipatory (warning signal of ecosystem change), display disturbance and changes over time (*i.e.*, well-documented model), and show low variability in terms of responses [26]. Numerous data in the literature provide evidence in support of *Neoceratium* meeting several of these conditions (Table 2); these are discussed below.

**Table 2:** *Neoceratium* features that match the prerequisites for ecological indicator validation.

Prerequisites for Ecological Indicators	*Neoceratium* Features
Easily measured	Quick identification within phytoplankton
	Ubiquitous, all year round present
Sensitive to stress	Sensitive to change in water temperature
Predictable response to stress (water warming)	Appearance of warm-water species
	Shift in seasonal pattern
	Northward extension range
	Increased abundance/prominence
Anticipatory response to change	Fast response to change because of short generation time
Well-known response to natural and anthropogenic changes	Well-documented and well-studied genus
	Profuse literature (biogeographical studies, presence/absence data, long-term series)
Low variability in response	Low variability in response at species level

Monitoring one or several *Neoceratium* species represents a quite inexpensive and easy measurement process. As previously detailed, *Neoceratium* cells are easily recognizable within phytoplankton samples by virtue of their typical morphology, size and dominance in the dinoflagellates fraction. In addition, their all year round presence and cosmopolitan distribution lend to a potential use as an indicator on a world-ocean scale and in various climatic situations. Unlike the monitoring required for other diatoms or dinoflagellates species which is often laborious, that of *Neoceratium* implies quite simple logistics. First, *Neoceratium* species are preferentially obtained by harvesting in a phytoplankton net which allows the rapid collection of a significant number of cells to observe. Second a classic light microscope and a low magnification (100×) are sufficient to perform taxonomic identification of *Neoceratium* taxa [27]. The identification of the majority of the thecate dinoflagellates is based on tabulation and necessitates fluorescent labeling of the theca or the delicate dissection of the cell. These time-consuming manipulations are not compatible for the monitoring of a species via the routine observations of cells. In contrast, the current taxonomic identification of *Neoceratium* is exclusively based on morphological criteria which mainly consider the global size and shape as well as the curvation of the horns [8]. Recently developed, well-illustrated and accessible websites aiming to help taxonomists and non-taxonomists identify phytoplanktonic species are valuable tools for promoting this kind of monitoring. One website in particular is dedicated to the identification of *Neoceratium* taxa at the species and infraspecies levels [28]. The use of such a website illustrating the infraspecies richness should prevent any confusion at the species level, and thus provide a reliable tool for the measurement of *Neoceratium* occurrence. Although phytoplankton have a short generation time, and should theoretically respond very quickly to environmental changes, their dynamics are driven by different ecological factors which results in large annual and inter-annual variability in terms of species assemblages [29]. This makes highlighting any possible change in composition related to ocean warming very arduous. Although globally present at quite low abundances, *Neoceratium* species show consistent all year round and year-to-year presence [4,27,30]. This particularity decisively provides consistent time-series which allow the detection of a significant response to any environmental change. The sensitivity of *Neoceratium* species to water temperature and how they respond to temperature increase may therefore permit the anticipation of the likely response of the genus to future ocean warming. Numerous studies have thus investigated the response of *Neoceratium* to warming. In terms of abundance, warming seems to have a positive effect on the *Neoceratium* species, as with other dinoflagellates [31]. In the laboratory, a recent experimental study focusing on the effect of warming on the transfer of carbon in the planktonic community highlighted that even a slight increase of temperature seemed to favor the development of *Neoceratium* species [32]. This trend is supported by the results of many large-scale investigations. One study analyzing Continuous Plankton Recorder data found that the abundance of several *Neoceratium* species increased in the North Sea during the post-90s while the central point of the spatial distribution pattern of *Neoceratium furca* moved northward as a likely consequence of climate change [33]. Phenological changes were also observed in the same area with an earlier occurrence of seasonal peak for several dinoflagellate genera, including *Neoceratium* [34]. Warming had also affected *Neoceratium* composition during the last century, as shown by a study conducted in the northwestern Mediterranean Sea, based on comparing old data gathered in the literature to new data obtained from analysis of recent samples [30]. Indeed, the warming of surface waters seemed to have subtly modified the seasonal assemblage of *Neoceratium* species in the Ligurian Sea, which tended to become closer to the assemblage characterizing the Tyrrhenian waters. In addition, a decrease in species richness was observed during the warm season in surface samples while it increased in vertical ones, suggesting that deeper localization may represent another possible phenological response of stenothermic taxa to warming. Clear patterns have thus been identified in the response of *Neoceratium* to increases in water temperatures, in terms of biomass, composition and phenology.

The choice of phytoplanktonic species like *Neoceratium* as ecological indicators is also relevant with regards the need to anticipate changes in the community structure and/or the environment due to climate change. Indeed, phytoplankton presents short generation times, which ensure their fast response to and thus our early detection of any modification or perturbation of the environment.

*Neoceratium* has been studied for decades and as such the literature provides a lot of information describing its response to natural perturbations and anthropic stresses. This is an important prerequisite to evaluate a target response to a specific change (here warming) from the established pattern of known responses [26]. Thus, diverse biogeographical studies characterizing the thermal ranges of occurrence of *Neoceratium* species and clustering them according to their biogeographical affinities [3,4,23,24] provide valuable tools to detect possible biogeographical changes due to warming. In addition, there exist many checklists that record the occurrence of dinoflagellate species including *Neoceratium* species based on the review of phytoplankton studies [5,35,36,37,38,39,40]. These constitute a very useful database to assess the appearance or disappearance of *Neoceratium* species in the different marine basins, or to identify patterns of biogeographical extension.

**Figure 2:**
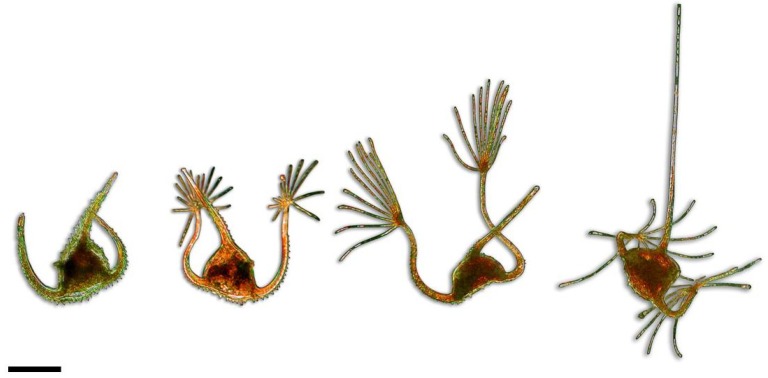
Variations of digitation configuration at the extremities of the antapical horns in *Neoceratium ranipes*. One dark period cell without digitations as opposed to three light period cells exhibiting daily-formed digitations. Bar scale 50 μm. Lugol fixed-cells.

Finally, an ecological indicator should preferentially show a low variability in response, in order to isolate the effective response to a change from the natural background variability. One may consider that *Neoceratium* does not really meet this criterion as variability of morphology is one of its features known to be influenced by several parameters, including water temperature. Some of these morphological changes reflect physiological processes that have, until recently, been completely ignored. For example, the successful cultures of *Neoceratium ranipes* revealed that the variations in the number and length of digitations at the extremities of the antapical horns (Figure 2) were the consequence of their deployment/retraction following a diurnal cycle [16]. While the presence and size of these appendages were previously considered as possible adaptations to improve the floatability in lower viscosity conditions, the results from this study suggest for the first time that these spectacular morphological changes could in fact reflect an adaptation of pigment content to light conditions, or a process to control sinking speed and circadian vertical migrations. This remarkable case and another on the seasonal cycle of *Neoceratium* taxa [27] thus confirm that additional parameters other than water temperature may influence the great variability observed in the *Neoceratium* genus. However, this variability may be mainly related to an intraspecific plasticity as was suggested by the first phylogenetic study of the genus [2]. Each sequenced species constituted a distinct clade in accordance with their morphological differences and was represented by a single sequence which could not express possible infraspecific polymorphism. At the species level, *Neoceratium* should therefore provide good indicators of water warming, at the condition that their infraspecific variability is identified, clearly documented and accessible to ensure a reliable taxonomic identification. A better illustration of this infraspecific variability in identification tools which still often neglect this aspect is first required [28]. More phylogenetic analyses including several sequences of both specific and infraspecific taxa would then confirm the status of morphologically distinct species and clarify the taxonomic level of variability.

## 4. Potential *Neoceratium* Metrics for Monitoring of Ocean Warming

While enriching the old bibliography, the recent advances in *Neoceratium* ecology and biology reinforce the relevancy of using *Neoceratium* species to monitor ocean warming. The genus could thus provide different kinds of metrics for use as ecological indicators that consider either the species assemblage or one target species.

First, monitoring the total abundance of *Neoceratium* taxa should constitute an easy-to-acquire measurement that reflects the change in phytoplankton assemblage. For instance, this metric could provide a good indication of dinoflagellate evolution with climate change since increasing temperatures have been shown to favor their net growth rate [41,42,43]. This can result in the shifting of phytoplankton composition and functioning, sometimes at the expense of other phytoplanktonic groups, like diatoms [31]. In the context of ecosystems management and health status monitoring, phytoplankton is usually employed as an indicator of nutrient conditions in aquatic and coastal ecosystems, the assessment of which is required by several legislations [44]. Currently, chlorophyll a constitutes the metric most commonly used to describe phytoplankton community evolution [44], since it provides a simple and integrative measure of phytoplankton biomass [45]. Nonetheless, this kind of indicator does not take into account heterotrophic species which play an important trophic role in the planktonic ecosystem [46]. Consequently, new metrics depicting phytoplankton composition are required by legislations such as the Water Framework Directive (WFD, 2000/60/EC) [47]. Assuming that *Neoceratium* dynamics would reflect those of other dinoflagellates characterized by the same ecophysiological requirements, a metric based on *Neoceratium* species abundance monitoring in coastal and marine ecosystems could meet such requirements.

Monitoring total *Neoceratium* assemblage on a regional scale may reveal a trend towards northward extension in response to regional warming. Indeed, since planktonic organisms are primarily subjugated by water masses and currents, the first and most obvious consequence of a change in their environmental conditions is the modification of their distribution range [48]. In fact, the latitudinal changes of distribution related to sea warming, and/or the change in seasonal patterns of abundances, have already been documented for other planktonic groups, e.g., copepods [49] and ciliates [50]. In Europe, a northward movement of species in open ecosystems in response to warming is expected, while in semi-enclosed ones (e.g., Black Sea and Mediterranean Sea), the decline of endemic species is likely to be compensated by the arrival of species from adjacent seas [51]. As an example, special attention could be given to the appearance in the western Mediterranean of warm-water *Neoceratium* (*N. breve*, *N. egyptiacum*, *N. reflexum*) that extended their distribution range from the eastern basin and the Atlantic with warming [30].

Monitoring the long-term trends in the richness of specific species can also constitute a good indicator of warming. In particular, some stenotherm species have been shown to disappear from surface waters in warmer conditions [30]. This type of metric would provide interesting insights into changes in phytoplankton diversity in response to temperature increase.

Surveying one or a small number of specific also is able to reflect a trend in the response to warming with the advantage of considerably limiting the monitoring effort. For such a survey, the selection of the target species should depend on geographical location and consider the species’ biogeographical characteristics as well as any local *Neoceratium* assemblage previously described. The thermal range of a species may differ from one basin to another. For instance, one recent study focusing on the annual cycle of *Neoceratium* in the Ligurian Sea underlined some discrepancies with the thermal ranges of occurrence previously described in biogeographical analyses conducted in other oceanic regions than the Mediterranean Sea, especially with regards to some warm-water species occurring in colder waters than expected [27]. The numerous checklists that have now been published should offer a useful basis on which to select appropriate species indicators according to location.

## 5. Conclusions

These recent data provide new insights into the diversity and taxonomic richness of *Neoceratium* and reinforce the interest in using this genus as a biological model in marine research studies. Finally, the monitoring of *Neoceratium* species as an indicator of ocean warming offers undisputable advantages including moderate counting effort as the only genus which is both cosmopolitan and perennial with easily recognizable cells for easy isolation from samples. In addition, current literature provides a rich database made up of regional species checklists and biogeographical resources. This biological model may also meet the requirements of new ecological indicators by reflecting the phytoplankton composition rather than being solely based on chlorophyll-derived biomass.

The development of digitization tools over the last few decades will also provide new perspectives for plankton surveys on ocean or even worldwide scales [52]. For instance, the FlowCAM (Flow Cytometer And Microscope), was designed to specifically detect and digitize particles in the size range of phytoplankton (2–3000 μm) [53]. The use of such an instrument will greatly benefit the analysis of very large numbers of samples required for a biogeographical study. It should also prove valuable in the detection and monitoring of phytoplanktonic indicators, such as *Neoceratium* species, in the context of modern oceanographic campaigns aiming to assess marine biodiversity on a global scale. One example would be the 2.5 year Tara Oceans expedition undertaken to explore the complex properties of the planktonic ecosystems [54].
